# Behavior of Cross Arms Inserted in Concrete-Filled Circular GFRP Tubular Columns

**DOI:** 10.3390/ma12142280

**Published:** 2019-07-16

**Authors:** Fang Xie, Ju Chen, Qian-Qian Yu, Xinlong Dong

**Affiliations:** 1 Faculty of Mechanical Engineering & Mechanics, Ningbo University, Ningbo 315211, China; 2 Department of Civil Engineering, Shaoxing University, Shaoxing 312000, China; 3 Department of Civil Engineering, Zhejiang University, Hangzhou 310058, China; 4 Department of Structural Engineering, Tongji University, Shanghai 200092, China

**Keywords:** concrete-filled, cross arm, displacement, GFRP, transmission

## Abstract

Fiber-reinforced polymer (FRP) materials nowadays have attracted much attention in both retrofitting of aged infrastructure and developing of new structural systems attributed to the outstanding mechanical properties. Extensive studies have been performed on concrete-filled glass FRP (GFRP) tubes for the potential application in piling, poles, highways overhead sign structures and bridge components. The new hybrid member also provides an alternative solution for traditional transmission structures. However, the connection between concrete-filled GFRP tubes and cross arms has not been fully understood. In this paper, an experimental study and theoretical analysis were conducted on the behavior of cross arms inserted in concrete-filled circular GFRP tubular columns. Steel bars with a larger stiffness in comparison with GFRP tubes were selected here for the cross arm to simulate a more severe scenario. The structural responses of the system when the cross arms were subjected to concentrated loads were carefully recorded. Experimental results showed that the concrete-filled GFRP tubes could offer a sufficient restraint to the deformation of the cross arm. No visible cracks were found on the GFRP tube at the corner of the cross arm where the stress and strain concentrated. Theoretical solutions based on available theories and equations were adopted to predict the displacement of the cross arms and a good agreement was achieved between the prediction results and experimental findings.

## 1. Introduction

Fiber-reinforced polymer (FRP) materials, having a high strength-to-weight ratio, good durability to corrosion and fatigue, as well as ease of installation, have been widely adopted in retrofitting of aged structures [1,2,3,4,5,6,7,8,9,10,11,12,13,14]. These advantages are also recognized in the development of new structural elements and systems. In recent years, extensive studies have been conducted on concrete-filled FRP tubes for the potential application of piling, poles, highways overhead sign structures and bridge components [15,16,17,18,19,20,21,22,23,24,25]. The hybrid system highlights the outstanding material properties of FRP, which provides a lightweight permanent formwork for concrete and improves the resistance to aggressive environmental conditions. When subjected to compression, the FRP tube offers confinement to the concrete core, increasing both the strength and ductility of the member. In addition, the concrete core could prevent or delay the FRP tube from buckling inward.

In Fam and Rizkalla [22], a comprehensive study on the structural behavior of concrete-filled glass FRP (GFRP) tubular beams, columns and beam-column specimens under bending, compression, and eccentric axial loading, respectively, was presented. Test variables including concrete-filled, laminate structure of the tube and reinforcement ratio, were considered. Analytical models were established to further investigate a wider range of parameters, such as the fiber orientation within the FRP tube, the thickness of the FRP tube, and the diameter of the central hole.

Mirmiran et al. [23] performed an experimental study on the effect of slenderness on the compressive behavior of concrete-filled GFRP tubular columns. As the slenderness ratio increased, the columns strength as well as the axial and hoop strains were significantly decreased, implying underutilized confinement. A theoretical analysis was developed for the prediction of the structural behavior and a slenderness limit of the hybrid columns was proposed.

In addition to the experimental investigation, numerical simulation also provides an effective way to solve engineering problems [26,27,28,29,30]. In Mirmiran et al. [26], the finite element method was adopted to analyze the structural behavior of FRP-confined concrete under the monotonic load and cyclic load. Fam and Son [27] developed a nonlinear finite element model to study FRP tubular poles partially filled with concrete subjected to flexure. The optimum concrete-filled length was mainly examined.

Regarding long-term durability of the concrete-filled FRP tube systems, recent research work was also conducted to give a comprehensive view of the wider application of the innovative structures.

Robert and Fam [31] exposed concrete-filled GFRP tubes in salt solution at 23, 40, and 50 °C for up to 365 days. By examining the hoop tensile strength of the tube before and after exposure, it was found that the most severe deterioration occurred to the specimens exposed to 50 °C, which lost 21% of the strength. The variation of microstructural and physical characterizations of the GFRP tubes showed that the degradation was mainly due to the presence of micro-cracks on the external surface of the aged tubes. A further prediction was carried out based on the Arrhenius theory.

More recently, Li et al. [32] investigated seawater and sea sand concrete-filled FRP tubes immersed in salt solution. After exposure in artificial seawater at 40 °C for up to six months, the seawater and sea sand concrete maintained its strength whereas the hoop strength of the FRP was noticeably decreased by 8% to 39%. The reduction of the FRP hoop strength consequently led to the decrease of strength and ultimate axial strain of concrete-filled FRP tubes, especially for basalt FRP and GFRP tubes. Carbon FRP tubes showed better resistance to the aggressive condition. A parameter was proposed to account for the environmental effect in the theoretical solution.

The structural performance of concrete-filled FRP tubes subjected to freeze-thaw cycles (−18 °C to 18 °C) was studied by Li et al. [33]. Monitoring of hoop strain of the tubes indicated possible cracks and debonding between GFRP and concrete as the number of freeze-thaw cycles increased. A significant decline of axial strength, modulus, and strain of the concrete-filled GFRP tubes was found after 56 freeze-thaw cycles, while the GFRP-steel tube system was more durable.

GFRP materials also provide an alternative solution for transmission structures [34,35], such as transmission towers or transmission poles, which are exposed to aggressive environmental attack. The application of pultruded GFRP materials could prevent or delay deterioration and corrosion of these infrastructure [36,37,38,39,40]. In addition, the installation feasibility is greatly improved in mountain terrain and marshes attributed to the light weight of the materials and consequently, the cost of transportation and erection is considerably reduced. The recommended practice for FRP products for overhead utility line structures is presented by the American Standard ASCE Manual No. 104 [41].

In Wang et al. [42], full-scale tests on an FRP composite casing in the reconstruction of a 380 kV power transmission line were conducted. The structural behavior of the joints, when loaded under tension and compression, was carefully described.

Godat et al. [43] presented a series of experimental studies on glass fiber pultruded sections in electricity transmission towers, i.e., (i) angle-section, square-section and rectangular-section specimens subjected to axial compression; (ii) I-section and W-section specimens tested under bending. Available design manuals and analytical equations were adopted to predict the critical buckling load. In addition, estimation of the economic cost was carried out for the optimum design of a transmission tower with 69 kV along a distance of 10 km.

Guades et al. [44] investigated the mechanical properties of pultruded GFRP tubes for the potential use of power pole cross arms. Additional ±45 glass fiber reinforcement was adopted for the components to provide a stronger structural behavior. It was found that the specimens approximately showed a linear elastic behavior. Finite element models were also developed to simulate the compressive and flexural behavior of the square tubes.

To the best knowledge of the authors, the connection between the concrete-filled GFRP tubular columns and cross arms has not been well understood which obstructs the wider application of the new materials in transmission structures. This paper presents an experimental study and theoretical analysis on cross arms inserted in concrete-filled circular GFRP tubular columns. The specimens were subjected to concentrated loads on both ends of the cross arm. Test results showed that the cross arms were well restrained in the columns and the deformation was relatively small. Strain and stress concentration was obviously observed on the GFRP at the corner of the cross arm whereas no visible cracks were found which implied good feasibility of this connection scheme. Theoretical prediction of the displacement of the cross arm was performed based on available theories and equations. This study extends the understanding of concrete-filled GFRP tubes with cross arms inserted and provides some useful suggestions for the hybrid structural members for the engineering practice of transmission structures.

## 2. Experimental Program

The experimental program was conducted in the Engineering Structural Lab at Zhejiang University. A total of six concrete-filled GFRP tubular columns were designed and tested.

### 2.1. Specimen Geometry and Dimensions

**Figure 1:**
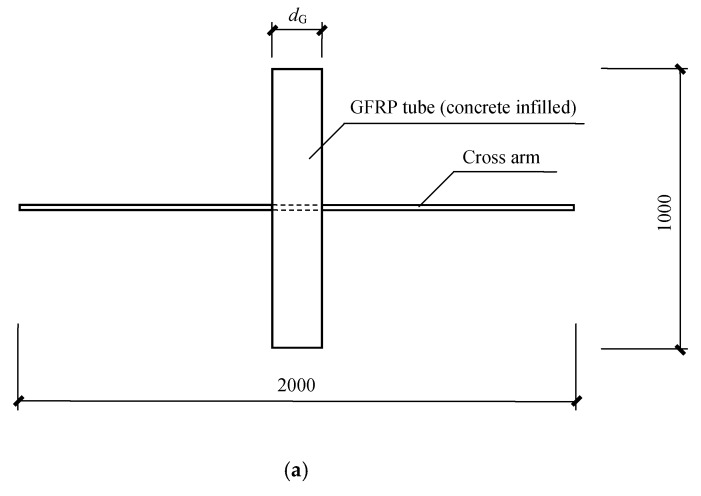

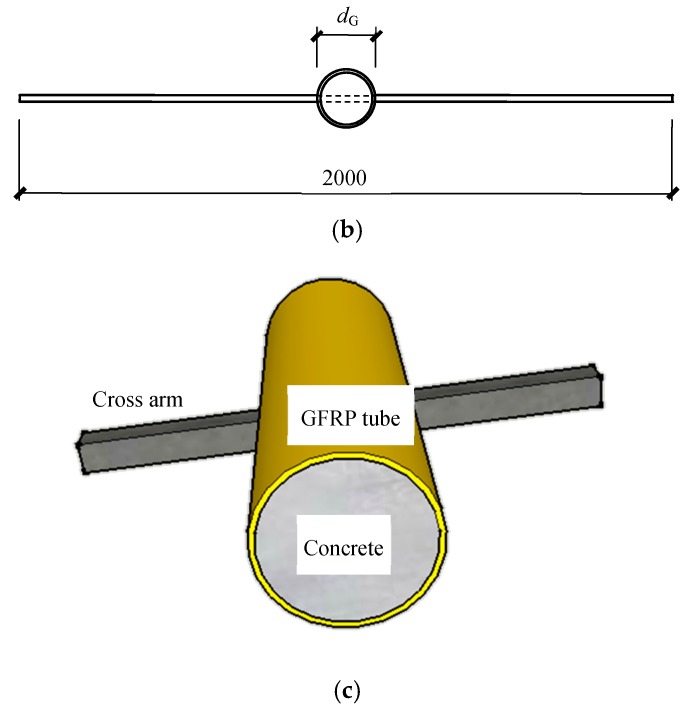
Specimen geometry and dimensions (unit in mm, not to scale): (**a**) Front view; (**b**) top view; (**c**) schematic diagram.

Figure 1 plots the geometry of the specimens and Table 1 gives detailed dimensions. The tubes had two core diameters of 180 mm and 300 mm, with the tube thicknesses of 10 mm and 12 mm, respectively. The columns had a height of 1000 mm and square steel bars were inserted to the mid-height of the columns through square holes to serve as cross arms in the transmission towers. Here, the steel bars rather than GFRP tubes were selected for the cross arm since the experiments were intended to investigate the unfavorable scenario where the steel bars had a larger stiffness and consequently a severer concern of the stress concentration at the corner. Five cross-sectional dimensions of the steel bars were adopted in the experimental program, i.e., 20 × 20 mm, 30 × 30 mm, 40 × 40 mm, 45 × 45 mm, and 60 × 60 mm.

### 2.2. Material Properties

The GFRP tubes were filament wound of unidirectional G-glass fibers at 45 angle (with respect to the longitudinal axis of the tube) and PET resin (Changshu Fengfan Power Equipment Co., Ltd., changshu, China). The mechanical properties of the GFRP tubes were provided by the manufacturer and are shown in Table 2.

The steel for the cross arms used in the tests was Q235b according to the Chinese Standard GB 50017-2003 Code for Design of Steel Structures [45]. The mechanical properties were obtained based on tensile coupon tests. The average yield strength, ultimate tensile strength, and Young’s modulus were 304 MPa, 475 MPa, and 204 GPa, respectively.

The tubes were filled with 22.7 MPa concrete based on the coupon tests [46] (28-day cubic compressive strength).

### 2.3. Specimen Preparation

**Figure 2:**
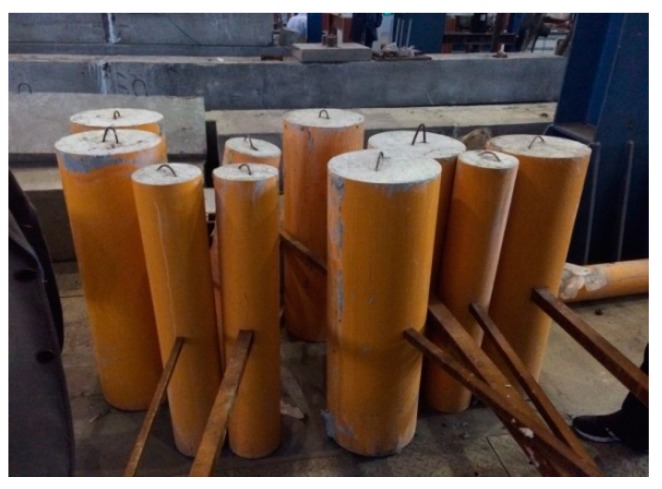
Specimens after preparation.

Two holes with the same dimension of the cross arm were first cut on each GFRP tube and a cross arm was placed in the center. Afterward, Grade C30 concrete was poured into the tubes and fully vibrated. All the specimens were cured in the room condition for 28 days. The specimens after preparation are shown in Figure 2.

### 2.4. Test Set-Up and Data Acquisition

**Figure 3:**
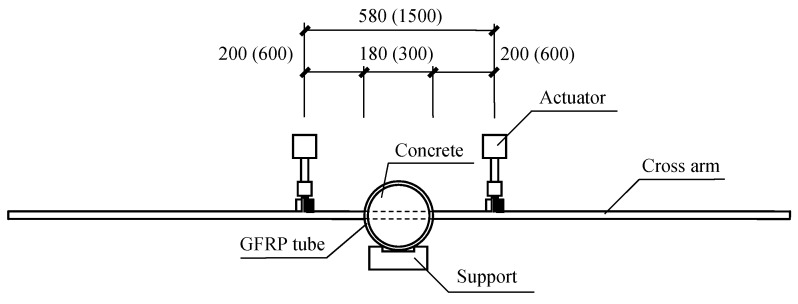
Test set-up (unit in mm, not to scale).

The tests were performed on a servo-hydraulic testing machine (Hangzhou Popwil Instrument CO., Ltd, Zhejiang, China) with a maximum loading capacity of 50 kN, as depicted in Figure 3. The scenario investigated in the current study was the effect of the load on the cross arms and columns transferred by wires of transmission towers, and therefore, the specimens were fixed on the floor by a notched concrete beam and loaded by an actuator on each end of the cross arm. The spans between the loading points were 580 mm and 1500 mm for the specimens with the column diameter of 180 mm and 300 mm, respectively. The figures out of and in the blanket shown in Figure 3 indicate the distances in the scenarios with the GFRP tube diameter of 180 mm and 300 mm, respectively.

The displacement control at a rate of 0.2 mm/min was adopted in the experiment. The test was stopped either when the steel of the cross arm yielded or the interaction hole on the GFRP tube cracked.

**Figure 4:**
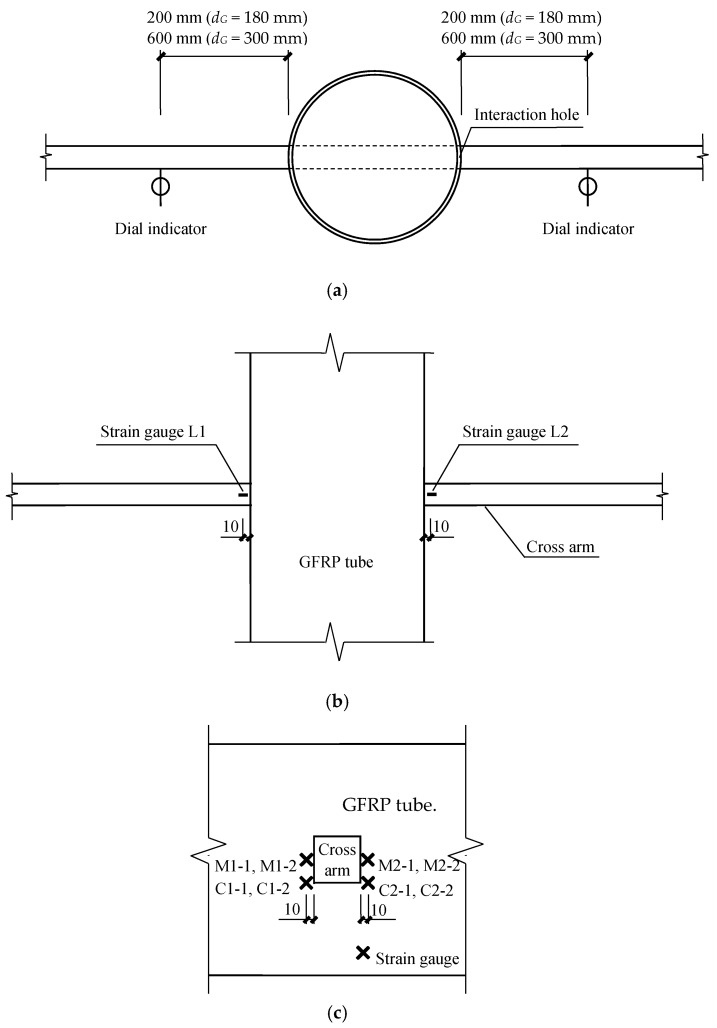
Data acquisition instruments: (**a**) Dial indicators on the cross arm; (**b**) strain gauges on the cross arm; (**c**) strain gauges on the GFRP tube.

Two dial indicators were mounted on the steel bar at the loading points to monitor the vertical displacement of the cross arm as shown in Figure 4a. The strain variation on the cross arm (at the loading side) was detected by a strain gauge attached to the cross arm near the GFRP tube, i.e., L1 and L2, as shown in Figure 4b. In terms of the strain on the GFRP tube during the test, two strain gauges were perpendicularly mounted to a certain position, which was along the fiber direction as depicted in Figure 4c. A total of eight strain gauges were attached, among which, four were in the middle of the cross arm (indicated as M1-1, M1-2, M2-1 and M2-2) and the other four were at the corner of the cross arm (indicated as C1-1, C1-2, C2-1 and C2-2).

## 3. Results and Discussions

### 3.1. Failure Mode

**Figure 5:**
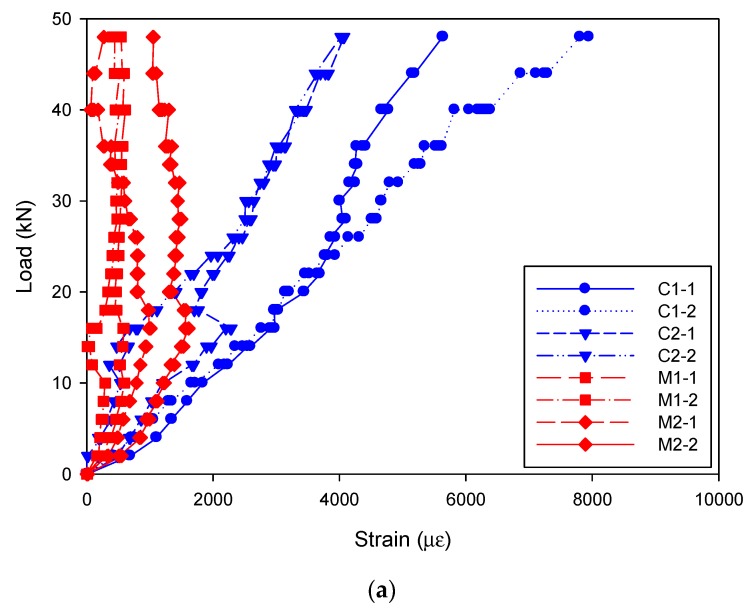

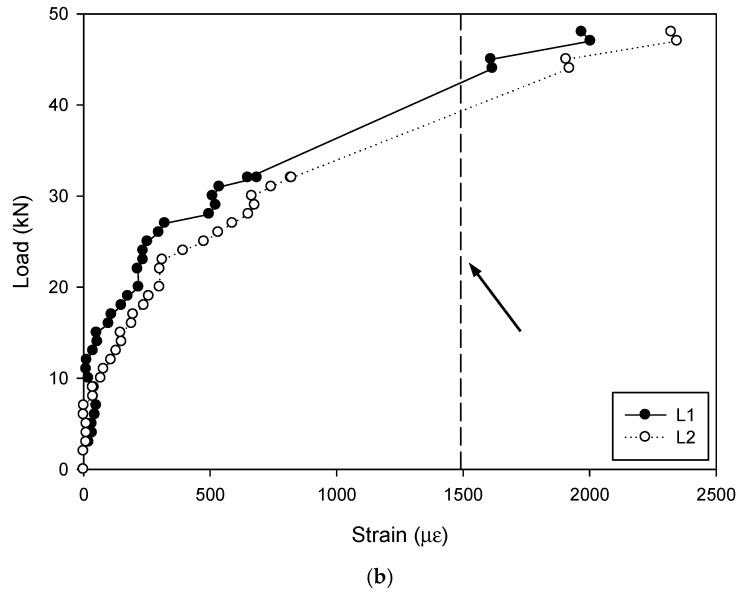
Typical load-strain curves of the specimen D150-40: (**a**) Strain gauges on the GFRP tube (compression); (**b**) strain gauges on the cross arm (tension).

During the loading process, no apparent phenomena were observed from the GFRP tube and concrete whereas an obvious deformation occurred to the cross arm. All the specimens failed due to yield of the steel bar rather than crack of the GFRP tube. Consequently, the strain and deformation on the cross arm were taken as the failure indicator. Figure 5 shows typical load-strain curves of the specimen D150-40, which gives a clear view of the failure process. As the load increased, the reading of the strain gauges on the GFRP tubes in Figure 5a increased as well. It was interesting to see that the strain on the GFRP tube at the corner of the cross arm was significantly larger than that in the middle position, indicating a severe stress concentration. However, the maximum strain reading at the ultimate load value found in the strain gauge C1-2 showed a value of 7948 με, which was apparently below the ultimate strain of the GFRP material (9310 με). Meanwhile, the maximum strain value observed for the strain gauges on the GFRP tube in the middle of the cross arm was 1614 με, equal to 63 MPa, which was maintained in a low stress level. The curves plotted in Figure 5b give a view of the nonlinear strain development on the cross arm versus the load. The strain first increased linearly with the load and generally transferred to a slowed increment. Based on the tested mechanical properties, the yielding strain of the steel was 1940 με, which indicated that the steel had reached its yield point.

### 3.2. Stain on the GFRP Tube

**Figure 6:**
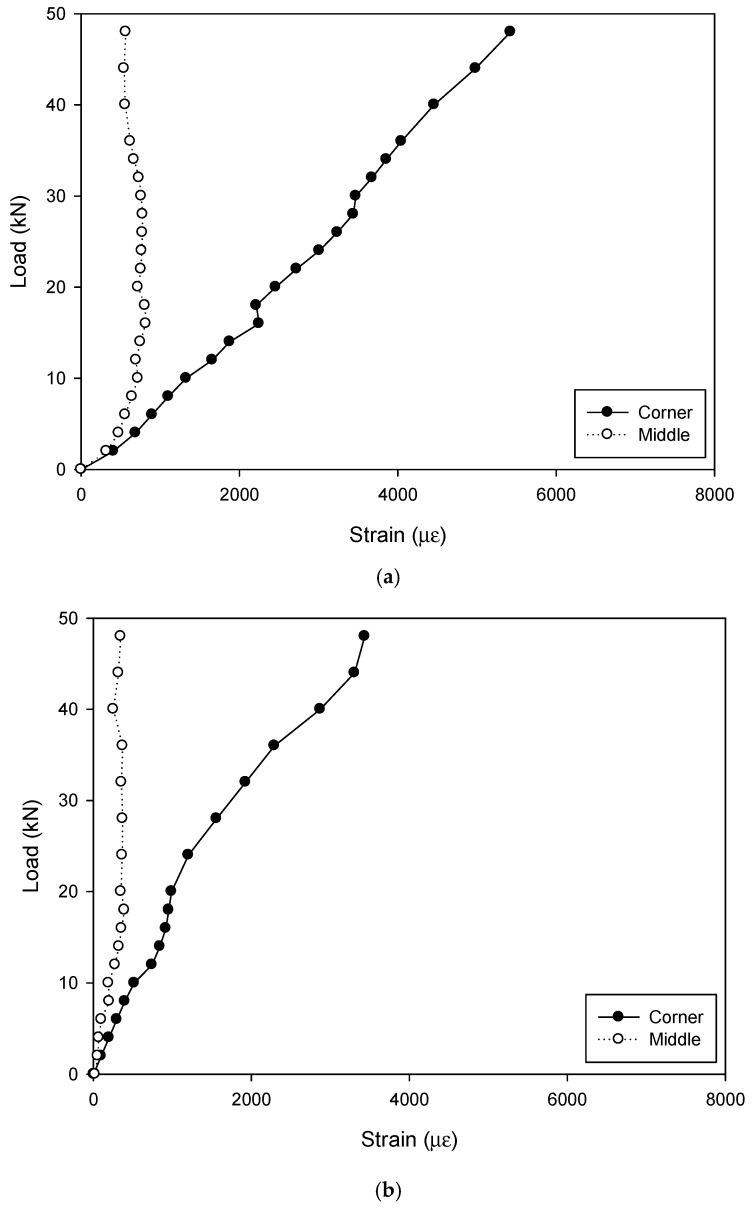

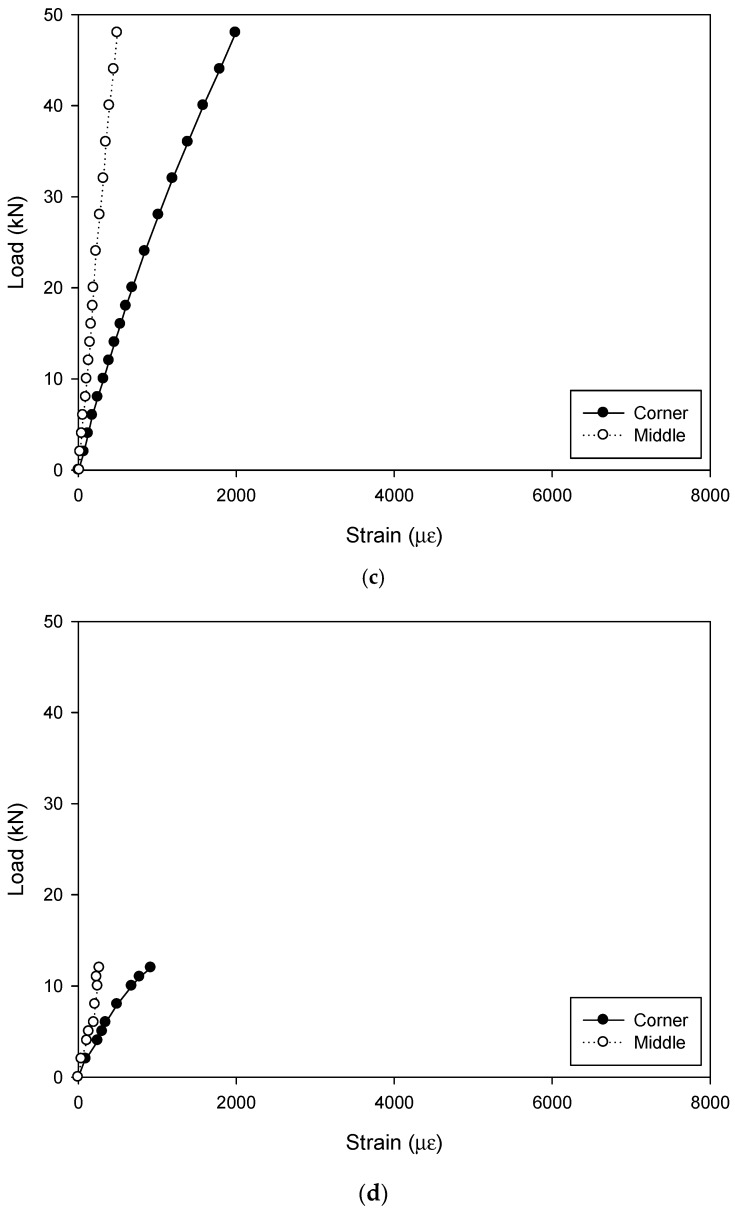

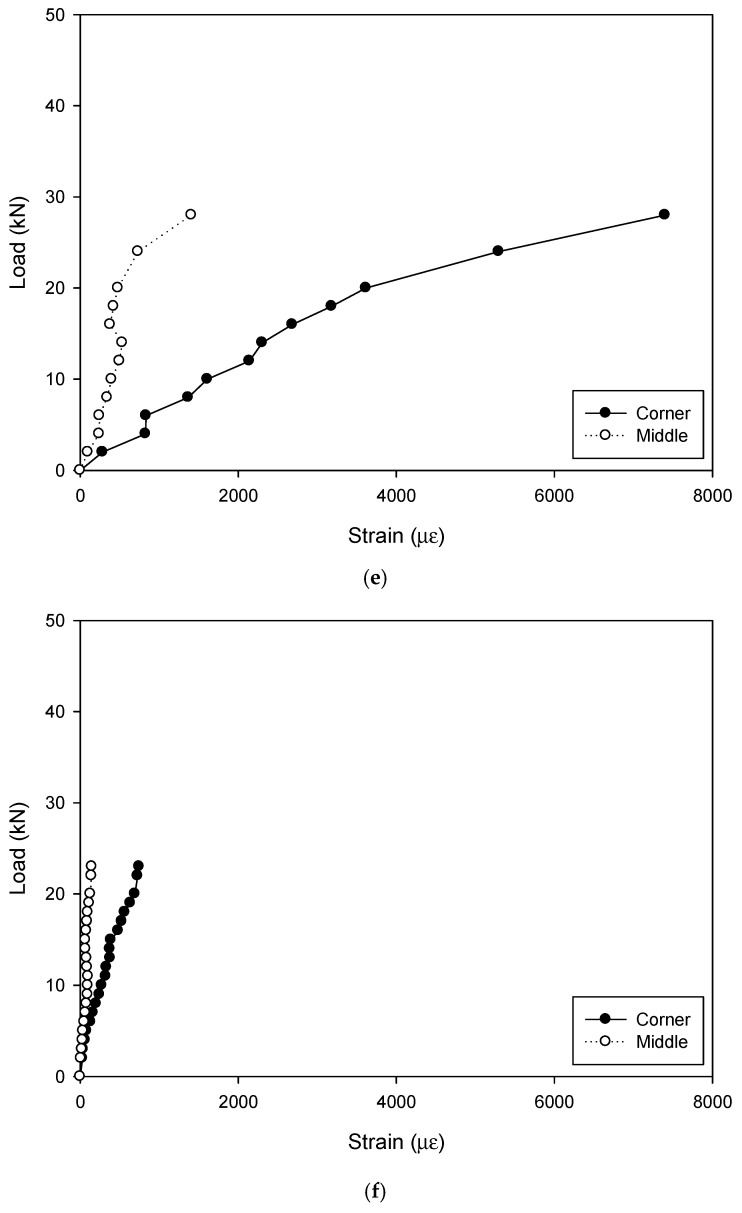
Comparison of the strains on GFRP tubes between at the corner and in the middle of the cross arms: (**a**) Specimen D150-20; (**b**) specimen D150-30; (**c**) specimen D150-40; (**d**) specimen D300-30; (**e**) specimen D300-45; (**f**) specimen D300-60.

In order to give an insight view of the structural performance of the GFRP tube, the strains of the four gauges at both the corner and middle of the cross arm were averaged and are compared in Figure 6.

It should be pointed out that, the ultimate loads diagrammed in Figure 6 for the specimens D150-20, D150-30 and D150-40 were approximately the same. The steel in the specimen D150-40 with the largest dimension of the steel bar had yielded at the ultimate load as shown in Figure 5b, and the steel in the specimens D150-20 and D150-30 reached the yielding point at a lower loading level. They were continuously loaded to the maximum load carrying capacity of the machine to give a chance to see the structural behavior after yielding. Differently, in the specimens with the GFRP tube diameter of 300 mm, the tests were stopped when the steel bars yielded.

Both the strains in the middle and corner of the interaction hole increased with the load while the increment at the corner showed a significantly larger rate in comparison with that in the middle part, indicating an apparent stress concentration at the corner.

In the engineering practice of transmission towers, the direction of the load applied to the cross arm is dependent on the wind direction and therefore, the load on the cross arm is definitely neither fixed nor symmetrical. Consequently, the GFRP at the corners of the cross arm bears more than that at other positions, which is much more prone to damage and calls for special attention. It is also suggested to apply local protection or strengthening to ensure durable performance.

**Figure 7:**
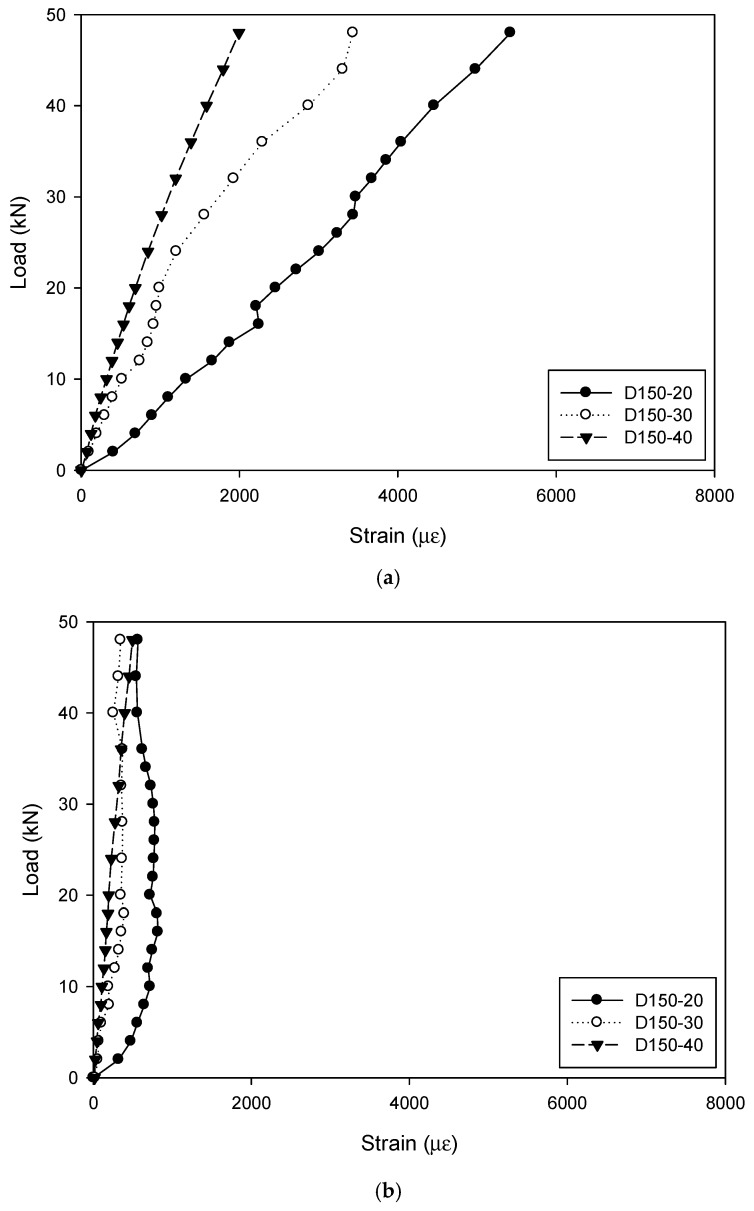
Strains of specimens with different dimensions of cross arms: (**a**) Strain on the GFRP tube at the corner of the cross arm; (**b**) strain on the GFRP tube in the middle of the cross arm.

Figure 7 compares the strains on GFRP tubes of specimens with different dimensions of cross arms. It is interesting to see that for the specimens with the diameter of GFRP tube of 150 mm, as the side length of the cross arm increased from 20 mm to 40 mm, the stiffness of the specimen was significantly improved. At the same load of 28 kN, the strain at the corner of the cross arm was generally decreased by 3439 με (D150-20) to 1562 με (D150-30) and 1019 με (D150-40), with a reduction percentage of 55% and 70%, respectively. However, in terms of the strain in the middle of the cross arm, it was always maintained in the low level, and the maximum value of 821 με occurred to the specimen D150-20. It was therefore concluded that the stress concentration on the GFRP tube at the corner of the cross arm was weakened in the specimen with a larger cross arm.

### 3.3. Displacement of the Cross Arm

**Figure 8:**
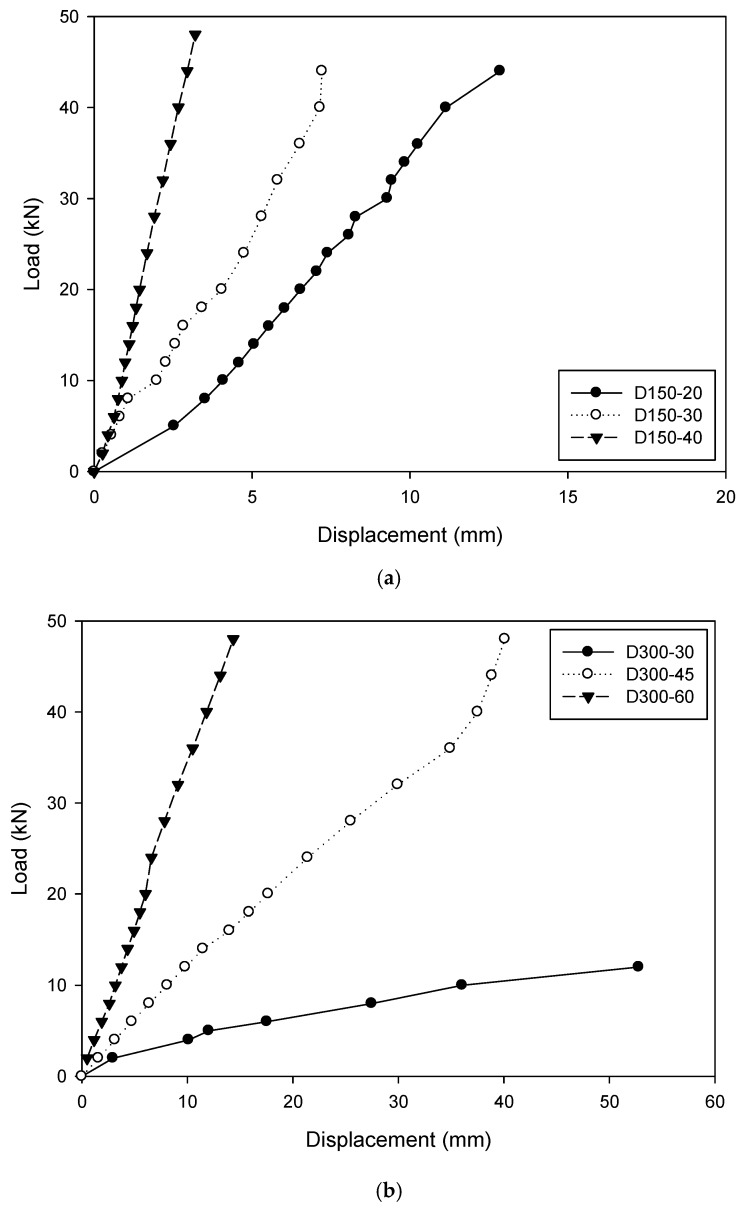
Vertical displacement of the cross arm at the loading point: (**a**) Specimens with the GFRP tube diameter of 150 mm; (**b**) specimens with the GFRP tube diameter of 300 mm.

For the vertical displacement of the cross arm at the loading points monitored during the tests, the results versus the load are plotted in Figure 8. It was clear to see that a larger dimension of the cross arm resulted in a stiffer cross section and consequently a smaller deformation.

Figure 8 compares the vertical displacement of the cross arm at the loading points between the specimens with the diameter of 150 mm and 300 mm. It was found that at the load of 28 kN, the displacement on the cross arm was decreased by 8.29 mm (D150-20) to 5.32 mm (D150-30) and 1.91 mm (D150-40), with a reduction percentage of 36% and 77%, respectively. For the specimens D150-30 and D300-30, which had the same dimension of the cross arm but two diameters of the GFRP tube, the considerable difference in the displacement on the cross arm was mainly attributed to the different spans between the loading points.

## 4. Theoretical Analysis on the Vertical Displacement of the Cross Arm

In engineering practice, the deformation of the cross arm is of critical importance for a transmission tower. Theoretical analysis was performed here to investigate the vertical displacement of the cross arm at the loading points. Solutions based on the mechanics of materials and elastic foundation beam theory were adopted and the results were compared with the experimental findings.

### 4.1. Theoretical Solution Based on the Mechanics of Materials

**Figure 9:**
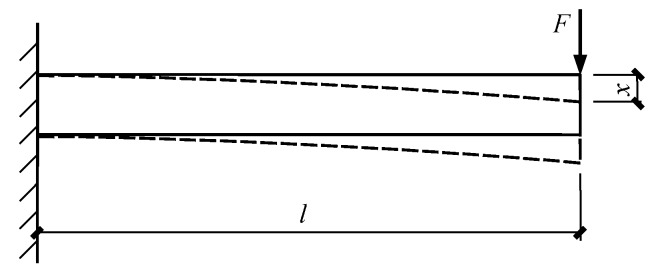
Schematic diagram of a cantilever beam.

Based on the experimental findings, the specimen failure depended on the strength and displacement of the cross arm. According to the mechanics of materials, the cross arm in such a concrete-filled circular GFRP tubular column was considered as a cantilever (Figure 9), whose displacement *x* subjected to the vertical load is expressed as Equation (1)

(1) $$x=\frac{Fl^{3}}{3EI}$$

where *F* represents the load applied by the actuator, *l* is the distance from the loading point to the fixed end, *E* is the elastic modulus of the cross arm and *I* is the moment of inertia of the cross arm.

### 4.2. Theoretical Solution Based on the Elastic Foundation Beam Theory

**Figure 10:**
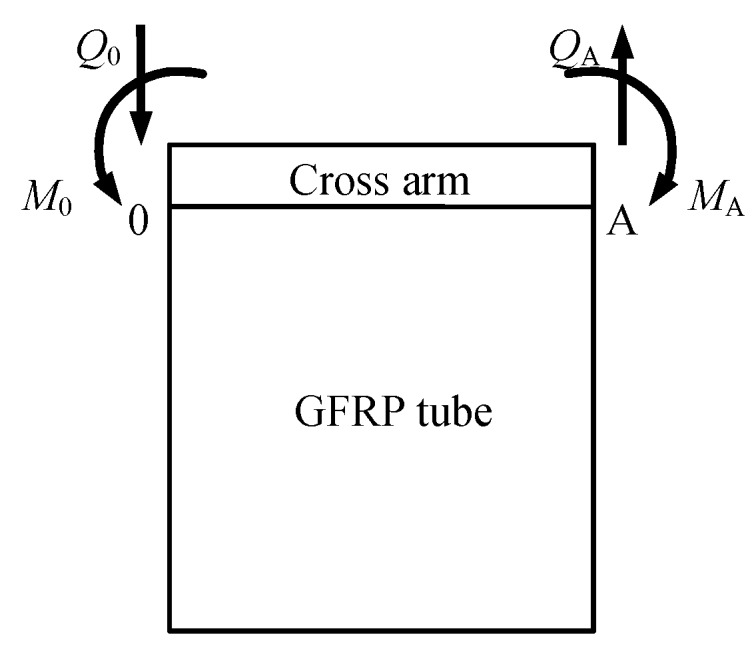
Schematic diagram of elastic foundation beam model.

**Figure 11:**
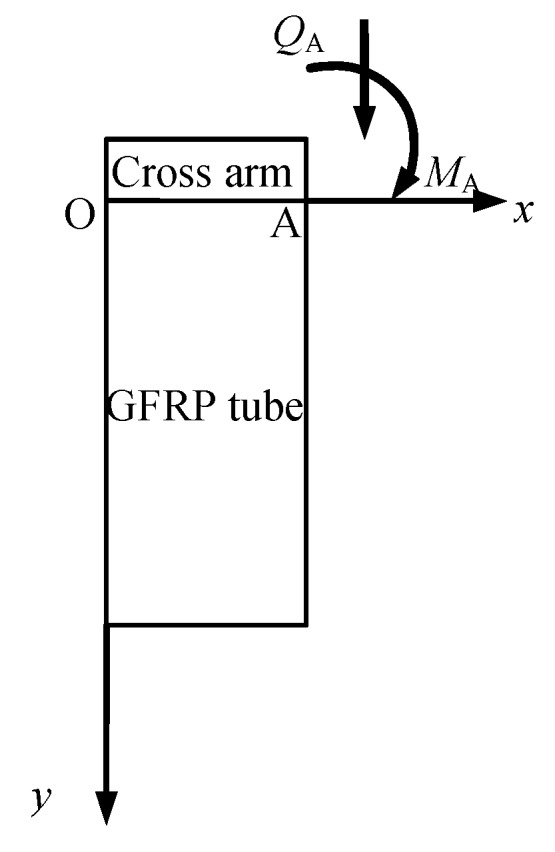
Half beam model.

According to the elastic foundation beam theory, the cross arm within the GFRP tube could be considered as laying on the concrete, as shown in Figure 10. The load applied on the cross arm was simplified as a concentrated force and moment. Only half a beam was developed in the model based on the symmetric boundary condition and loading scenario (Figure 11). The special solution of the flexural differential equation of a beam on the elastic foundation is expressed by Equations (2) to (5) [47].

(2) $$y=y_{0}\varphi_{1}+\vartheta_{0}\frac{1}{2\alpha }\varphi_{2}-M_{0}\frac{2\alpha^{2}}{bk}\varphi_{3}-Q_{0}\frac{\alpha }{bk}\varphi_{4}$$

(3) $$\vartheta =-y_{0}\alpha \varphi_{4}+\vartheta_{0}\varphi_{1}-M_{0}\frac{2\alpha^{3}}{bk}\varphi_{2}-Q_{0}\frac{2\alpha^{2}}{bk}\varphi_{3}$$

(4) $$M=y_{0}\frac{bk}{2\alpha^{2}}\varphi_{3}+\vartheta_{0}\frac{bk}{4\alpha^{3}}\varphi_{4}+M_{0}\varphi_{1}+Q_{0}\frac{1}{2\alpha }\varphi_{2}$$

(5) $$Q=y_{0}\frac{bk}{2\alpha^{2}}\varphi_{2}+\vartheta_{0}\frac{bk}{4\alpha^{3}}\varphi_{3}+M_{0}\alpha \varphi_{4}+Q_{0}\varphi_{1}$$

where *y* is the deflection, *ϑ* is the rotation angle, *M* is the bending moment, *Q* is the shear force, *φ*_1_ = ch*αx*cos*αx*, *φ*_2_ = ch*αx*sin*αx* + sh*αx*cos*αx*, *φ*_3_ = sh*αx*sin*αx*, *φ*_2_ = ch*αx*sin*αx* − sh*αx*cos*αx*, *α*=(*bk*/4*EI*)^1/4^, *b* is the width of the cross arm, *k* is the foundation coefficient, taken as 800 × 10^4^ kN/m^3^, *E* is the elastic modulus of the cross arm, taken as 206 GPa.

In this scenario, the boundary conditions at points “O” and “A” are described by Equations (6) to (7).

(6) $$\left\{ \begin{array}{l} y_{0}=0\\ \vartheta_{0}=0 \end{array}\right.,\;\mathrm{at}\;\mathrm{point}\;“O”$$

(7) $$\left\{ \begin{array}{l} M_{A}=-M=-Fl\\ Q_{A}=-P=-F \end{array}\right.\;\mathrm{at}\;\mathrm{point}\;“A”$$

At point “A”, *x* is equal to half of the diameter of the GFRP tube, which is 75 mm and 150 mm corresponding to the two groups of specimens.

Substituting all the known parameters to Equations (2) and (3), the value of *Q*_0_ could be subsequently derived. Afterward, the rotation angle *ϑ* was calculated with the available *Q*_0_. Eventually, the vertical displacement at the loading point of the cross arm was obtained.

### 4.3. Comparison of the Cross Arm Displacement between Theoretical Solution and Experimental Findings

**Figure 12:**
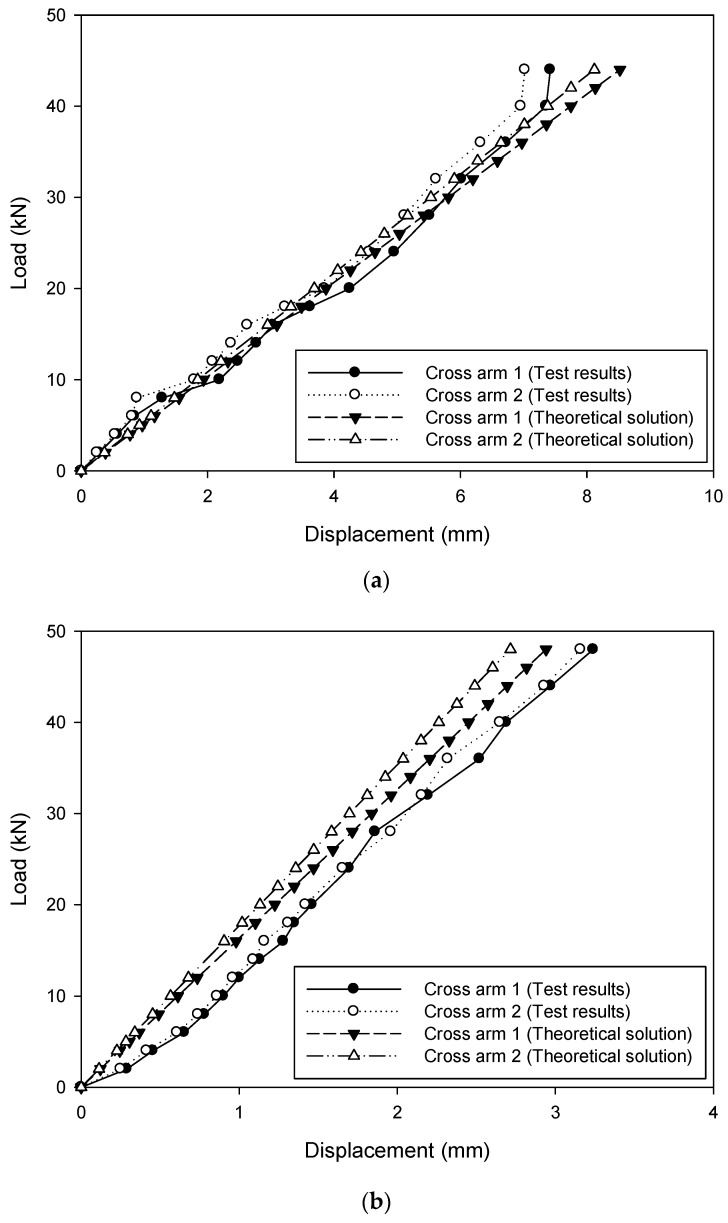

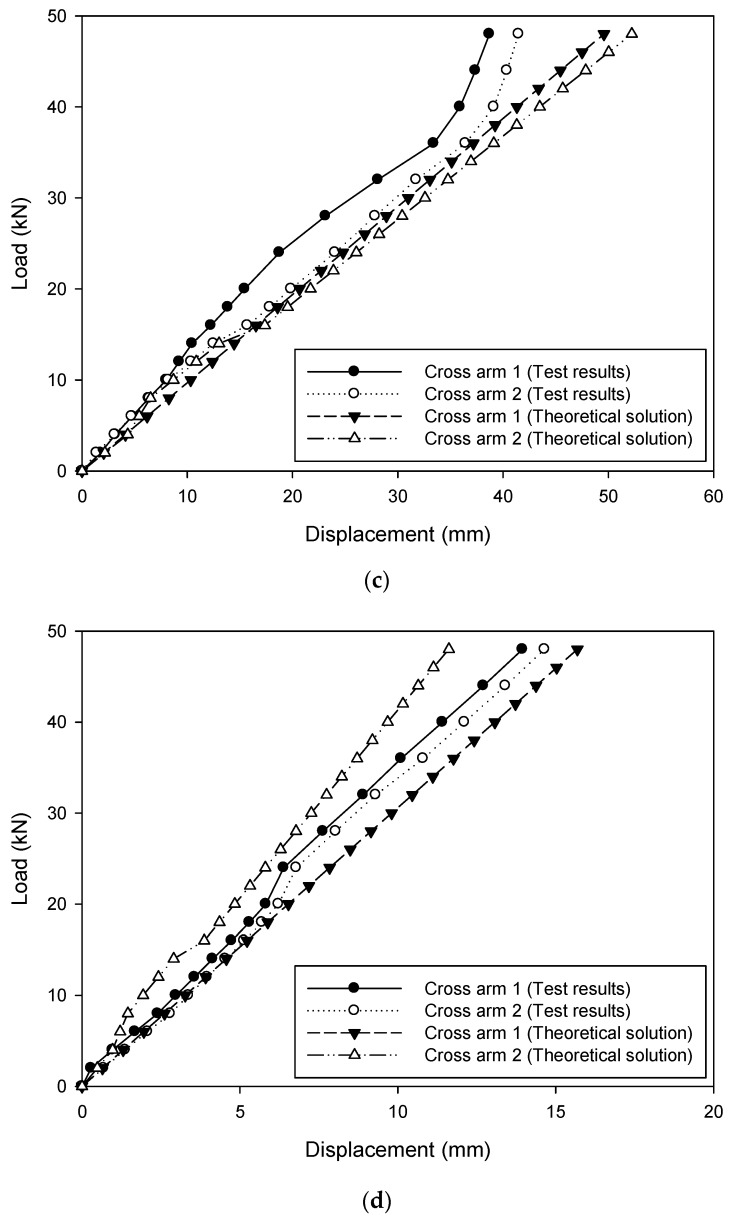
Comparison between the theoretical solution and experimental results: (**a**) Specimen D150-30; (**b**) specimen D150-40; (**c**) specimen D300-45; (**d**) specimen D300-60.

Vertical displacement of the cross arm at the loading points based on the theoretical solutions and experimental results for typical four specimens are compared in Figure 12. The horizontal and vertical axes represent the displacement and load, respectively. The legend symbols cross arm 1 and cross arm 2 indicate the reading of the deformation of the steel bar at the loading points. A good agreement was clearly observed. It was, therefore, demonstrated that both the theoretical solutions based on the mechanics of materials and elastic foundation beam theory can be successfully used for estimating the structural response of the cross arms inserted in concrete-filled circular FRP tubular columns. Consequently, the assessment process could be adopted in the design of transmission structures.

## 5. Conclusions

In this study, a total of six concrete-filled GFRP tubular columns with cross arms inserted were tested under concentrated loading applied to the cross arms. The test parameters included the dimensions of the GFRP tube and the steel bars acted as the cross arm, as well as the loading span. Based on the limited test results, the following observations can be made and conclusions drawn.

The connection between the concrete-filled GFRP column and the cross arm adopted in this study showed a promising response. The rotation of the cross arm was well restrained by the columns and the stress level on the GFRP tube was relatively low.

Considerable strain and stress concentration was found on the GFRP tube at the corner of the cross arm. As the dimension of the cross arm decreased, more severe strain and stress concentration occurred. However, no visible cracks were found in the current test program. All the specimens failed due to yield of the steel bar rather than crack of the GFRP tube, which showed great promise for the engineering practice.

The displacement of the cross arm was well predicted based on the mechanics of materials and the elastic foundation beam theory. The estimation was therefore reliable to assess the deformation of the hybrid structural element.

## Figures and Tables

**Table 1:** Specimen dimensions.

Specimen	Diameter of the GFRP Tube *d_G_* (mm)	Wall Thickness of the GFRP Tube (mm)	Cross-Sectional Dimension of the Cross Arm (mm)
D150-20	180	10	20 × 20
D150-30	180	10	30 × 30
D150-40	180	10	40 × 40
D300-30	300	12	30 × 30
D300-45	300	12	45 × 45
D300-60	300	12	60 × 60

**Table 2:** Material properties of GFRP tubes.

GFRP Tube	Water-Absorption Rate (%)	Degree of Cure (%)	Density (kg/m^3^)	Ultimate Tensile Strength (MPa)	Elastic Modulus (GPa)
*d_G_* = 180 mm	0.15	95.9	2066.5	342.6	36.8
*d_G_* = 300 mm	0.14	92.7	1987.1	371.9	39.2
